# A Systematic Review of the Relation between Complementary Feeding and Children’s Development

**DOI:** 10.1007/s13668-025-00692-7

**Published:** 2025-08-30

**Authors:** Alice Di Prete, Amy T. Galloway, Claire Farrow, Francesca Bellagamba, Elsa Addessi

**Affiliations:** 1 Sapienza Università di Roma, Rome, Italy; 2 Department of Psychology, Appalachian State University, Boone, North Carolina, USA; 3 School of Psychology and Institute for Health and Neurodevelopment, Aston University, Birmingham, UK; 4 CNR, Istituto di Scienze e Tecnologie della Cognizione, Roma, Italy

**Keywords:** Complementary feeding, Infant diet, Infant development, Baby-led weaning, Weaning approach

## Abstract

**Purpose of Review:**

The aim of this systematic review is to assess the relationship between two complementary feeding features (diet quality and feeding approach) and some aspects of infant development (cognitive development, social cognition, and language).

**Recent Findings:**

Recent reviews have explored many aspects of complementary feeding, such as the age at which first foods are introduced, child’s micronutrient status, weight and health outcomes. However, there appears to be a knowledge gap in two areas that are potentially relevant for developing a healthy diet: the quality of the diet and the type of complementary feeding approach. Furthermore, reviews on complementary feeding tends to rely heavily on research from Western countries (also known as WEIRD countries), failing to provide a global perspective on infant development.

**Summary:**

Positive significant relationships were found between diet quality and (i) cognitive development (five studies), (ii) language development (six studies), (iii) social cognition (three studies), (iv) general development (six studies), and between approach and language development (two studies). Although a substantial number of findings suggest a significant relationship between diet quality and child development (20), some findings were non-significant (17), signaling that more research is needed in this field.

## Introduction

### Rationale

Early childhood is a sensitive period for growth, development, and well-being of infants [1] and can be seen both as a time of tremendous opportunities for neurodevelopment and a time of great vulnerability [2]. Early brain development is very rapid and calorie-demanding [3], and rapidly growing organs are more vulnerable to damage if critical nutritional substrates that support this growth are not adequately provided [4]. This vulnerability includes both a defined time limit after which repair of an atypically developed system is no longer possible [5] and broader periods of time when a developing system is particularly responsive to shaping [6].

Many developmental changes occur during early childhood and having a healthy diet is an important protective factor, while adverse dietary experiences and malnutrition can have long-term negative effects (lower IQ scores, reduced academic success, and behavioral dysregulation [7]). During this developmental period, it appears to be important that children learn to accept foods and beverages in order to establish long-term healthy dietary patterns [8]. Complementary feeding is the process of providing foods in addition to breast milk or formula when milk alone is no longer adequate to meet a child’s nutritional requirement. This phase generally starts when the infant is 6-month-old (with some countries anticipating it to 4 months of age, although this practice is discouraged by the Word Health Organization [9]) and has long-lasting consequences for the individual well-being [8]– [10].

Many aspects of complementary feeding have been explored in recent reviews, such as the age of introduction of complementary feeding [11–14], complementary feeding in preterm infants [15]– [16], the relationship between complementary feeding and micronutrient status [17], weight outcomes [18–20], and health outcomes [21], and the efficacy of complementary feeding interventions to promote children’s health and growth in low and middle income countries [22]– [23]. However there seems to be a gap in knowledge in two specific areas, which are potentially important for developing a healthy diet: the quality of the diet, and the type of complementary feeding approach.

As for diet quality, a commonly used measure is the Dietary Diversity Score (DDS), defined as the number of food groups consumed by the child over a certain period of time [24]. The food groups considered are usually animal sourced foods (eggs, dairy, fish, and meat), nuts/pulses/seeds, fruits/vegetables, and starchy staple foods (grains such as wheat, maize, and rice) [9]. Some studies have considered more sub-categories of the DDS depending on their aims (sweets and/or snacks [25]– [26]; breast milk [27–29]), and on their geographical location (tubers and plantains in Congo [27], berries and liver paste in Norway [25]). The World Health Organization, in its later recommendations about complementary feeding, advises the consumption of animal-sourced foods, fruits and vegetables on a “strong, low certainty evidence,” and the consumption of pulses, nuts, and seeds on a “conditional, very low certainty evidence” [9]. These recommendations signal that there is the need to better understand how the consumption of certain foods during complementary feeding affect child development. This is particularly true for young children from developing countries, where international sources report that energy intake during the complementary feeding period is significantly lower than recommended [30–34]. Globally, only 28% of children aged 6–23 months meet the minimum dietary diversity (MDD) indicator level. The lowest rates were found in South Asia, West and Central Africa, and Eastern and Southern Africa (about 25%), and the highest rates in Latin America and the Caribbean (62%). In East Asia, the Pacific, the Middle East and North Africa, only 39%-36% of children aged 6–23 months met the MDD level [34].

We were able to find only one systematic review on the relation between diet quality during complementary feeding and developmental milestones [35]. This study considered a very wide age range (from 4 months to 18 years of age) during which development could be influenced by many factors other than complementary feeding. It also excluded studies conducted in LMIC (Low- and Middle-Income Countries). However, it is important to include children from both WEIRD (Western, Educated, Industrialized, Rich, and Democratic [36]) countries and the Majority World [37]– [38] to have a global view of development, including societies that have been neglected by research although numerically representing the majority of Earth’s population. Indeed, 91% of participants in developmental studies come from WEIRD countries [39], though they represent only 5% of the World population [40].

The caregiver’s approach to complementary feeding also has important implications for child development. In many Western countries, infants are traditionally introduced to solid foods for the first time as spoon-fed pureed [9], with a gradual transition to coarser-textured finger foods and family foods around 12 months of age [41]– [42]. This approach is usually defined as Parent-Led Weaning (PLW) [43]. However, over the past 20 years, alternative approaches to PLW have been introduced, such as Baby-Led Weaning (BLW). This approach was first proposed in the UK [44] and involves infants attending family meals, being offered finger foods and allowed to self-feed [45–47]. BLW, and other similar approaches that allow the child some degree of independence during meals (as “on-demand complementary feeding” in Italy [48]), have been linked to advantages in some areas of development, such as self-regulation in food intake [49], participation in family meals [51], motor development [52]– [53], language development [54]– [55], and caregiver’s responsiveness to child’s cues [56–58]. To our knowledge a few non-systematic reviews have explored the characteristics and the recommendations concerning the BLW method [45, 47, 59–61]. However, no systematic review has ever been conducted on the relationship between the complementary feeding approach and infant development considered as an outcome, rather than on the health risks and benefits of the different complementary feeding approaches [62–65].

### Objectives

The aim of this systematic review was to assess the relationship between complementary feeding features, namely diet quality and complementary feeding approach, and infant development (cognitive development, social cognition^1^ and language) in both Western and Majority World societies.

## Methods

### Eligibility Criteria

We planned to include experimental (randomized and non-randomized controlled trials) and observational studies with an internal comparison group (cohort prospective and retrospective) involving infants with typical development, from 6 months to 6-years of age. Intervention studies and studies on children with atypical development (e.g., premature births), health conditions (e.g., HIV, anemia), and who consumed specific nutritional supplements, were excluded. Primary outcomes were cognitive development, social cognition, and language development. We chose to investigate diet quality and the complementary feeding approach as, from a preliminary literature search and considering the most recent World Health Organization guidelines on complementary feeding [9], it appeared that these aspects are especially relevant during the complementary feeding phase. As developmental outcomes, we selected cognitive development, social cognition, and language development as they yielded the largest number of papers in a preliminary literature search. [See Table 1]

### Information Sources

Four online databases (Psycinfo, Pubmed, Scopus and Web of Science) were searched for the first time on January 24th, 2024, with no restrictions on language or publication date. Further searches were carried out within the reference list of the articles selected. No unpublished studies were sought. Searches were re-run prior to the final analysis on February 21st, 2025.

### Search Strategy

The following key words were used: (“Complementary feeding” OR “Weaning phase”) AND (“Language development” OR “Cognitive development” OR “Social cognition”). Data was extracted from each database in RIS format to enable the entry in Rayyan (a semi-automated software tool which helps researchers screen, assess, organize and filter large quantities of articles when writing a review) [67]. There were no restrictions on language or publication date. No supplementary filters were applied. We did not use any natural language processing or text frequency analysis tools nor any translation tools.

### Selection Process

One reviewer (ADP) applied the eligibility criteria and selected studies to include in the systematic review and another one (EA) checked the decisions. A third person (FB) impartially intervened to resolve disagreements. All decisions were recorded and revised through Rayyan [67], an online tool that allows to easily sort articles and extract data for reviews.

### Data Collection Process

ADP screened titles and abstracts and later the full texts of potentially eligible studies, and EA verified the accuracy of data extraction (for 20% of the articles). A third person impartially intervened to resolve disagreements (FB). No contact with the original study investigators was necessary. All data was managed and revised through Rayyan [67], and selected papers were also reported in an Excel spreadsheet. Rayann allowed to automatically eliminate double records, but we also refined this process manually not to lose any important data. Information to consider in data collection and data extraction was selected according to the PICOS approach.

### Data Items

Major outcomes were cognitive development, social cognition, and language outcomes measured by means of standardized observational tests and parental self-report (any measure was eligible for inclusion). The exposure considered was the quality of the child diet and the type of complementary feeding approach used by the parents, which were assessed as well through standardized observational tests or parental self-reports. We also recorded the age of the participants, the country in which the study was carried out, the sample size and the percentage of female participants involved.

### Study Risk of Bias Assessment

Quality assessment was performed using the MMAT tool [68], a checklist for concomitantly appraising and describing studies included in systematic reviews that include qualitative, quantitative, and mixed methods studies. Only moderate- to high-quality studies (score from 3 to 5) were considered for inclusion. A complete description of the scale can be found in [68]. Assessment was done at the study level. ADP made the quality assessment of data, and EA verified that the checklist was followed correctly. A third person (FB) impartially intervened to resolve disagreements. No automated tools were used in this process.

### Synthesis Methods

Collected data was synthetized using descriptive analysis. No minimum number of studies for the data synthesis was considered. ADP synthesized the data, and EA checked the synthesis. A third person (FB) impartially intervened to resolve disagreements. Information to consider were related to study generalities, objectives, methods, participants, outcomes, and results according to the PICOS approach.

**Table 1:** The table reports a synthesis of the steps described in the method section

Inclusion criteria	Experimental and observational studies with an internal comparison group involving infants with typical development, from 6 months to 6 years of age
Exclusion criteria	Intervention studies and studies on children with atypical development, health conditions, and who consumed specific nutritional supplements
Databases	Psycinfo, Pubmed, Scopus and Web of Science
Key words	(“Complementary feeding” OR “Weaning phase”) AND (“Language development” OR “Cognitive development” OR “Social cognition”)
Major data outcomes	Cognitive development, social cognition, and language outcomes measured by means of standardized observational tests and parental self-report questionnaires
Secondary data outcomes	Age of the participants, country in which the study was carried out, sample size and percentage of female participants involved

## Results

### Study Selection

A total of 1099 articles resulted from searching the four databases (Pubmed = 35, Scopus = 1006, Psycinfo = 5, Web of science = 53) during the initial search (January 24, 2024). A total of 1279 articles resulted from searching the four databases (Pubmed = 39, Scopus = 1176, Psycinfo = 5, Web of science = 59) during the second final search (February 21, 2025).

**Fig. 1:**
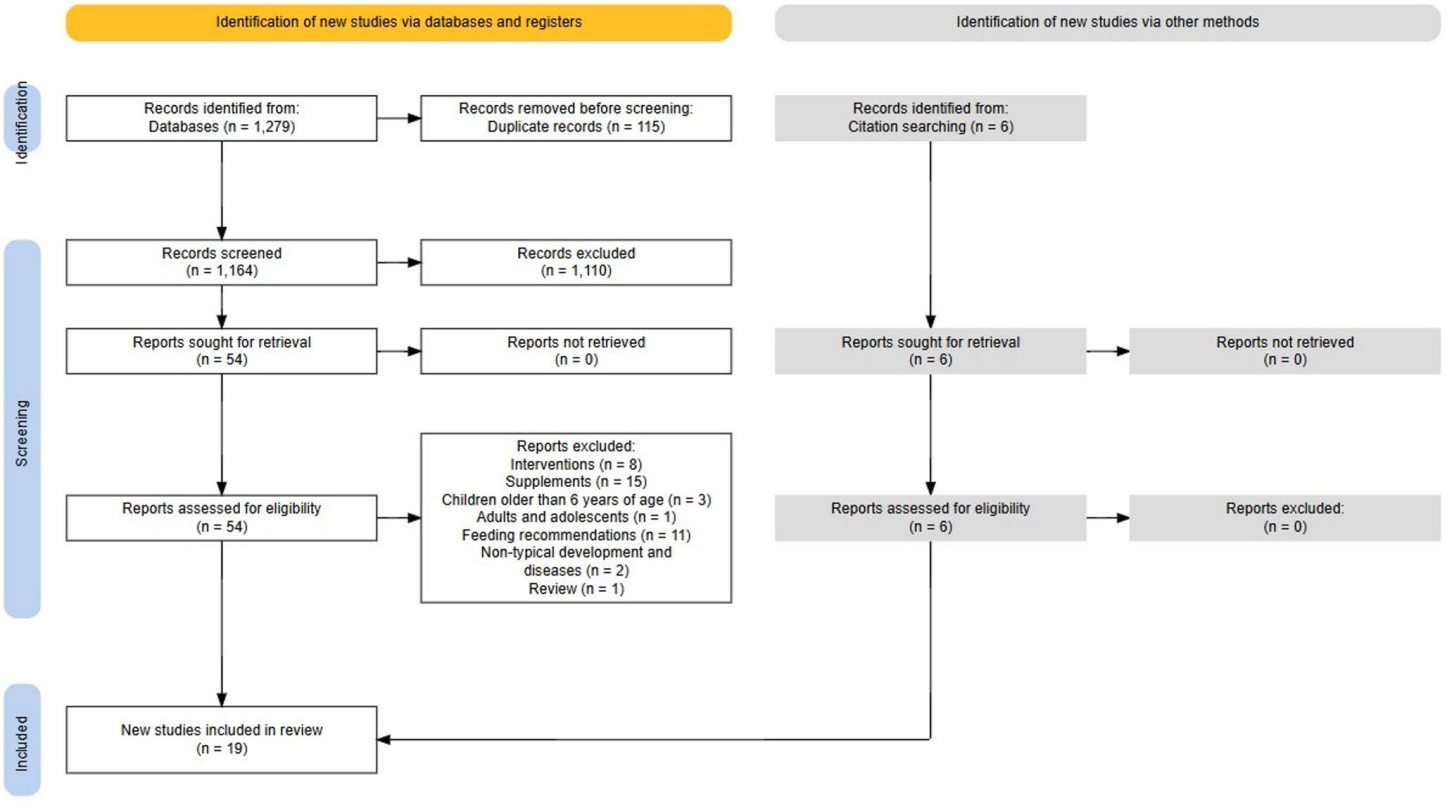
PRISMA flow diagram [76]

115 duplicates were automatically excluded by Rayann and manually checked by ADP. 1110 papers were excluded after reading the abstract and title (304 articles on non-typical development and diseases, 200 articles on breastfeeding, 180 reviews and meta-analysis, 160 articles on feeding recommendations, 85 articles on supplements, 70 articles on interventions, 70 articles on adults and adolescents, 15 study protocols, 13 books or book chapters, 8 articles on the development of an instrument, 4 articles on animals and one article on children older than 6 years of age). After reading the complete remaining texts, 41 more papers were excluded (8 articles on interventions, 15 articles on supplements, 3 articles on children older than 6 years of age, one article on adults and adolescents, 11 articles on feeding recommendations, 2 articles on non-typical development and diseases and one review). From the bibliography of the remaining 13 articles, we selected 6 further papers ( [52] from [54]; [69] from [70]; [71] from [72]; [73] from [28]; [74] from [28]; [75] from [28]), for a total of 19 articles. [See Fig. 1]

### Study Characteristics

Of the 19 studies selected, 10 were longitudinal cohort studies, 7 were cross-sectional studies, one was a longitudinal cohort study with a cross-sectional component, and one was a secondary data analysis of a cluster-randomized controlled trial [See Table 2 for details].

Seven studies were from Europe (four from the UK, one from Norway, one from Italy and one from Portugal), five studies were from Africa (two from Kenya, one from Congo, one from Egypt, and one from Uganda), five studies were from Asia (three from Nepal, one from Singapore and one from China) and two studies were from the Americas (one from the USA, and one from Haiti). Study participants were almost equally divided between males and females (max females 71% - min females 43%) and their number ranged from a minimum of 58 to a maximum of 1534. 13 papers out of 19 were published in the last five years [See Table 2 for details].

Diet quality was measured in 16 out of the 19 selected studies (one study used two instruments). Six studies used the 24-h recall method, four studies used the Food Frequency Questionnaire (FFQ) [77–82], two studies weighted the food consumed, and two studies used the household DDS tool [83]. Other instruments used were the observation by a professional, the probed oral recall method and the food diary method [used in one study each; See Table 2 for details].

The complementary feeding approach was measured in only three studies out of the 19 selected, and it was measured with a survey [43, 46, 84]. [See Table 2 for details].

The cognitive outcome was measured in 13 out of the 19 selected studies (four studies used two methods). Five studies used the Ages and Stages Questionnaire (one study used the Extended Ages and Stages Questionnaire [EASQ; 85], whereas four studies used the ASQ third edition [85]– [86]), seven studies used the Bayley Scales of Infant and Toddler Development (three studies used the first edition [87]; one study used the second edition [88]; two studies used the third edition [89]– [90], and one study used the fourth edition [91]. Other instruments were the NIH Toolbox for Assessment of Neurological and Behavioral Function (NIHTB-CB) ( [92] one study), the Kaufman Brief Intelligence Test-2 (KBIT-2) ( [93] one study), the Raven’s colored progressive matrices ( [94] one study), the Piaget-based Infant Psychological Development Scale (IPDS) ( [95] one study), the Wechsler Pre-School and Primary Scale of Intelligence third edition (WPPSI-III) ( [96] one study) [See Table 2 for details]. Some of these instruments and methods measure cognitive development in a general wide range sense (WPPSI-III, Bayley, Bayley-II, Bayley-III, Baylet-4, KBIT-2), while others have a narrower focus: AQS-3 and EASQ (problem solving), NIHTB-CB (fluid cognition), Raven’s colored progressive matrices (non-verbal cognition), IPDS (object permanence, objects as means and foresight).

Social cognitive development (as defined in the Section “Objectives”) was measured in five out of the 19 selected studies, which used the Ages and Stages Questionnaire (one study used the EASQ [97]; four studies used the ASQ third edition [85]– [86]) [See Table 2 for details].

Language outcomes were measured in 16 out of the 19 selected studies. Four studies used the Ages and Stages Questionnaire (one study used the EASQ [97]; four studies used the ASQ third edition [85],– [86]), three studies used professional observation, two studies used the MacArthur Communicative Development Inventory (MCDI) [98] and two studies used mother reports [99]. Other instruments used were the Developmental Neuropsychological Assessment (NEPSY) ( [100] one study), the Bayley Scales of Infant and Toddler Development-III (Bayley-III) ( [89] one study) [See Table 2 for details]. Most instruments measured language development as a composite total score (ASQ-3, EASQ), while others considered only some aspects of it (MCDI and Bayley-III considered language comprehension and production, whereas NEPSY considered verbal fluency). Mother-reported measures were utterance of first words in a study and total consonant and vowel production in another one. Observations by professionals reported the number of spontaneous toddler vocalizations.

Finally, two out of the 19 selected studies used the Ages and Stages Questionnaire-3 [101] without differentiating within development areas, but only considering the total score (a combination of communication/language, gross motor, fine motor, problem-solving and personal-social skills scores). Five studies considered the total score of the Ages and Stages Questionnaire [85, 86, 97, 102–104] in addition to the subscale scores [See Table 2 for details].

**Table 2:** Study characteristics and key findings (PICOS approach)

Reference	Study design	Country	Sample size	Age (months)	Female %	Diet quality and feeding method measure	Development outcome measure	Outcomes
Webber et al. 2021	Cross-Sectional	UK	131	8–24	71	Questionnaire about complementary feeding experiences	MCDI	Children exposed to a more independent approach at the onset of the complementary feeding period were more likely to have higher scores in language comprehension and production. Children who were offered family foods were more likely to have higher language comprehension and production scores.
Addessi et al. 2021	Cross-Sectional	Italy	1245	6–12	46.2	Survey about the complementary feeding approach	Mother-reported utterance of first words	No feeding variables were associated with the age of first words utterance.
Blomkvist et al., 2019	Cross-Sectional	Norway	212	12	47.6	Food Frequency Questionnaire	ASQ-3	Dietary intake of fish, fruits and vegetables was associated with higher developmental scores.
Farrow et al., 2024	Cross-Sectional	UK	58	Under 2 years of age	37.25	Family Mealtime Coding Scheme	Observations MCDI	Observed infant self-feeding was positively associated with the number of infant vocalisations
Miller et al., 2020	Longitudinal (L) cohort with a Cross-Sectional (CS) component	Nepal	629 (CS-sample) + 269 (L-sample)	CS: 23–66 L: 6–18	49.7% (CS-sample) + 46.8% (L-sample)	24-h recall method	ASQ-3	In the cross-sectional sample, greater consumption of eggs or dairy products was associated with a higher total Ages and Stages Questionnaire-3 score. In the longitudinal sample, greater egg consumption, cumulative dietary diversity and animal sourced food scores (at age 6–18/23–66 months) were associated with increased odds of high total Ages and Stages Questionnaire-3 score (at age 23–66 months).
Thorne-Lyman et al., 2019	Longitudinal cohort	Nepal	305	14 23–38	47.9	24-h recall method	ASQ-3	Each additional day of consuming at least 4 food groups was associated with a 33% reduction in the odds of low total Ages and Stages Questionnaire-3 score. Food consumption from animal sources was associated with 36% lower odds of having a total Ages and Stages Questionnaire-3 score and 32% lower odds of having a low communication score. Dairy consumption was associated with 42% lower odds of a low total development score and 33% lower odds of a low communication score. Frequent vegetable consumption was associated with low scores for the communication and personal-social scales. The consumption of animal sourced foods, vegetables, and fruits (but not of processed foods) was associated with a higher total development score.
Gale et al., 2019	Longitudinal cohort	UK	241	6–12 48	46.1	Food Frequency Questionnaire	WPPSI-II NEPSY	Children whose diet was characterized by a higher consumption of fruit, vegetables, and home-prepared foods had a higher full-scale and verbal IQ at 4 years of age.
Wilk et al., 2022	Longitudinal cohort	USA	61	12–60	52	Food Frequency Questionnaire	Bayley-4 NIHTB-CB	No significant relationship between dietary intake of beef (6–12 months) and cognitive function (1–5 years).
George et al., 2021	Cross-Sectional	Congo	117	6–23	55	24-h recall method	EASQ	Minimum dietary diversity was associated with a significantly higher combined Extended Ages and Stages Questionnaire score, communication score and personal social score.
Morgan et al., 2004	Longitudinal cohort	UK	144	4–24	48	Food diaries	Bayley-II	No association between meat intake (4–20 months) and mental developmental scores (22 months).
Cunha-Rodrigues et al., 2023	Cross-Sectional	Portugal	212	12–36	51.9	24-h recall method	Bayley-III	Girls with a higher energy contribution form unprocessed foods and with an above median minimum dietary diversity score had a higher neurodevelopment score; no significant association were observed in boys.
Toh et al., 2023	Longitudinal cohort	Singapore	484	6–12 24, 54	46.9	24-h recall method	KBIT-2 Bayley-III	Higher fat intake (6–12 months) was associated with higher receptive language scores (24 months) but with lower expressive language scores. Higher carbohydrate intake (6–12 months) was associated with higher receptive language scores (24 months). No significant associations were observed with neurodevelopment at 54 months.
Iannotti et al., 2016	Longitudinal cohort	Haiti	583	6–11 18–23	54.8	The household DDS tool	Mother-reported total consonant and vowel count	Early dietary diversity and consumption of specific foods (eggs and oils) increased the odds of later better language outcomes at months 18–23.
Sigman et al., 1991	Longitudinal cohort	Kenya	83	18–30 60	55.4	Observation by a professional	Bayley-I Raven colored progressive matrices test	Food intake (fat and animal protein) at months 18–30 was associated with better cognitive skills in 5-year-old children.
Wachs et al., 1993	Longitudinal cohort	Egypt	153	18–23 24–30	50.3	Probed oral recall Weighing of food	Bayley-I IPDS Observation	Toddler energy and protein and fat intake (18–30 months) predicted general cognitive development (24–30 months).
Pokharel et al., 2023	Longitudinal cohort	Nepal	701	6–18 24	52.1	Food Frequency Questionnaire	ASQ-3	Significant positive association between any animal sourced food consumption at 18 months of age and total Ages and Stages Questionnaire-3 score at 24 months of age.
Sigman et al., 1989	Longitudinal cohort	Kenya	110	18–30	51.8	Weighing of food	Bayley-I	Food intake (total kcalories, animal protein and fat intake (18–30 months) was related to the total amount of verbalization (30 months).
Zhao et al., 2020	Cross-Sectional	China	1534	6–23	43	24-h recall method	ASQ-3	Meeting the Minimum dietary diversity (MDD) was associated with a 39% lower risk of developmental delays. There was a significant association between MDD and reduced likelihood of developmental delays in problem-solving and personal social subscales (6–23 months). An inverse dose–response relationship was observed between the number of food groups consumed and the risk of developmental delays (6–23 months).
Kakwangire et al., 2021	Secondary data analysis of a cluster-randomized controlled trial	Uganda	385	6–8 20–24	48	The household DDS tool	ASQ-3	No significant association was found between the Dietary Diversity Score (6–8 months) and development domains (20–24 months).

### Risk of Bias in Studies

Risk of bias assessment was performed by using the MMAT tool [68]: three studies received a high-quality score (5), seven studies received a moderate-high quality score (4), and nine studies received a moderate quality score (3) [See Table 3 for details]. No studies were excluded for having a moderate-low (2) or low (1) quality score. Assessment questions are reported in the previous “Study risk of bias assessment” paragraph and more details are provided in [68].

**Table 3:** MMAT scores

	Screening questions		Non-randomized studies					MMAT score
	S1	S2	Q1	Q2	Q3	Q4	Q5	1–5
Webber et al., 2021	Y	Y	N	Y	Y	Y	Y	4
Addessi et al., 2021	Y	Y	N	N	Y	Y	Y	3
Blomkvist et al., 2019	Y	Y	N	Y	Y	Y	Y	4
Farrow et al., 2024	Y	Y	N	Y	Y	Y	Y	4
Miller et al., 2020	Y	Y	Y	N	N	Y	Y	3
Thorne-Lyman et al., 2019	Y	Y	Y	N	N	Y	Y	3
Gale et al., 2009	Y	Y	N	Y	N	Y	Y	3
Wilk et al., 2022	Y	Y	Y	Y	N	Y	N	3
George et al., 2021	Y	Y	N	Y	Y	Y	Y	4
Morgan et al., 2004	Y	Y	Y	Y	Y	Y	Y	5
Cunha-Rodrigues et al., 2023	Y	Y	Y	Y	N	Y	Y	4
Toh et al., 2023	Y	Y	Y	N	Y	Y	Y	4
Iannotti et al., 2016	Y	Y	Y	Y	Y	Y	Y	5
Sigman et al., 1991	Y	Y	N	Y	N	Y	Y	3
Wachs et al., 1993	Y	Y	N	Y	Y	N	Y	3
Pokharel et al., 2023	Y	Y	N	Y	N	Y	Y	3
Sigman et al., 1989	Y	Y	N	N	Y	Y	Y	3
Zhao et al., 2020	Y	Y	Y	Y	Y	Y	Y	5
	Screening questions		Randomized controlled trials					MMAT scores
	S1	S2	Q1	Q2	Q3	Q4	Q5	1–5
Kakwangire et al., 2021	Y	Y	Y	Y	N	Y	Y	4

## Results of Individual Studies

### Diet Quality and Cognitive Development

Out of the 13 studies that explored the relationship between diet quality and cognitive development, eight found no significant associations [26–28, 70, 75, 105–107], whereas five studies [29, 71–74] found a significant positive association (in one study for females only [72]). Specifically, infants (between 6 and 12 months) with a higher consumption of fruit, vegetables, and home-prepared foods had higher full-scale IQ at 4 years of age [71]. A higher fat and animal protein consumption in 18-30-month-old infants was associated with better cognitive skills at 24–30 months of age [74] and at 5 years of age [73]. Minimum dietary diversity (MDD) was associated with a higher score in the ASQ problem-solving subscale in 6–23-month-old infants [29]. Girls (between 12 and 36 months) with a higher energy contribution of unprocessed or minimally processed foods and with an above median MDD score had higher odds of achieving a higher neurodevelopment score [72].

### Diet Quality and Social Cognition

Out of the five studies that explored the relationship between diet and social cognition, two found no significant associations [70, 107], while three found a significant positive association [27, 29, 105]. A more frequent vegetable consumption was associated with a better score on the ASQ personal-social subscale in 23–38-month-old infants [105]. Minimum dietary diversity (MDD) was associated with a higher personal-social score in 6-23-month-old infants in both [27] and [29] studies.

### Diet Quality and Language Development

Out of the 10 studies that explored the relationship between diet and language development, five found no significant associations [29, 70, 71, 74, 107], four found a significant positive association [27, 75, 105, 108] and one found both a negative and a positive significant associations [28]. According to Thorne-Lyman [105], greater intake of animal-sourced food (ASF), dairy, and vegetable were associated with lower odds of having a low communication score in 23-38-month-old infants. Food intake (total kilocalories, animal protein and fat) in 18-30-month-old infants was related to the total amount of verbalization when infants were 30 months old [75]. Children whose diet in infancy (between 6 and 12 months) was characterized by a higher consumption of fruit, vegetables, and home-prepared foods had higher verbal IQ at 4 years of age [71]. Higher fat and carbohydrate intake in 6-12-month-old infants was associated with higher receptive language score at 24 months [28]. MDD was associated with a higher ASQ-3 communication score in 6-23-month-old infants [27], and both MDD and the consumption of eggs and oils increased the odds of better language outcomes in 6-11- and 18-23-month-old infants [108].

### Diet Quality and General Development

Out of the seven studies that explored the relationship between diet and general development (a combination of communication/language, gross motor, fine motor, problem-solving and personal-social skills scores), one study found no significant associations [70], while six studies found a significant positive association [25, 27, 29, 69, 105, 107]. Dietary intake of fish, fruits and vegetables was associated with higher total neurodevelopmental scores in 12-month-old infants [25]. In the cross-sectional sample of Miller et al. [69], 23-66-month-old infants with greater consumption of eggs or dairy had reduced odds of low general development score (ASQ). In the longitudinal sample of the same study, egg consumption and cumulative Dietary Diversity Score (DDS) and Animal Sourced Food (ASF) scores (in 6-18- and 23-66-month-old infants) were associated with reduced odds of low total ASQ developmental score at 23–66 months of age. ASF consumption in 18-month-old infants was also positively associated with total ASQ developmental score at 24 months of age [107]. According to Thorne-Lyman et al. [105], each additional day of consuming at least 4 food groups was associated with a 33% reduction in the odds of a low total ASQ developmental score in 23-38-month-old infants. Animal food sources, dairy and vegetable/fruits (but not processed food) consumption was also associated with a significant lower odd of having a low total ASQ score in 23-38-month-old infants. Minimum dietary diversity (MDD) was associated with a higher total development score in 6-23-month-old infants according to both [27] and [29]. Moreover, according to Zhao et al. [29], there was an inverse dose–response relationship between the number of food groups consumed and the risk of developmental delays in 6-23-month-old infants.

### Complementary Feeding Approach and Child Development

Out of the three studies that explored the relationship between the complementary feeding approach and child development, one found no significant associations on the utterance of the first words [52], while two found a significant positive association on language production and comprehension [54]– [55]. Addessi et al. [52] did not find a significant relationship between any of the features of the on-demand complementary feeding approach (namely self-feeding, finger-food feeding, and family-food feeding) and the age of first word utterance in 6-to-12-month-old infants, probably because the typical age of first word production is around 13 months of age [109]. In contrast, in Webber et al.’s [54] study, eight to 24-month-old infants who, at the onset of the complementary feeding period, were exposed to a feeding method that allowed more independence, were more likely to have higher language production and comprehension scores. Moreover, parents who reported offering their children family foods more often (an important characteristic of Baby-Led Weaning) were more likely to have children with higher language production and comprehension scores [54]. In Farrow et al. [55], a more infant-led approach to complementary feeding appeared to bring benefits for child language; more specifically in children under 24 months old (mean age = 14 months, SD = 4.15) observed infant self‐feeding was positively associated with the number of observed infant vocalizations.

## Discussion

This systematic review explores the existing literature on the relationship between two important aspects of complementary feeding, namely diet quality and type of complementary feeding approach, and their impact on several aspects of infant development. We included children aged between 6 months and 6 years old, with the intent of considering potential long-term outcomes. To enlarge the number of articles sampled, we did not limit our research to a specific publication period, to a socio-economical group, or to a geographical area. Furthermore, this review had no limitations with regard to outcome measures: we selected any measure available which also included self-report and non-validated instruments (or instruments that used non-local validation norms). This led to some “moderate” MMAT scores (3) [See Table 3 for details] that however could still be accepted according to our initial PROSPERO protocol (based on [68] guidelines). Although the lack of limitations allowed us to retrieve an acceptable number of papers to write an original review, it also led to partially divergent results.

Only five studies that explored the relationship between diet and cognitive development found significant positive outcomes [29, 71–74]. If we analyze the studies that led to non-significant outcomes in this area of development, we can see that three studies [26, 106]– [107] only considered meat or animal sourced food consumption (excluding many other food categories that could influence cognitive development) and the remaining studies [27]– [28, 70, 75, 105] were carried out on participants who were mostly highly to moderately malnourished or stunted. So, while dietary diversity and the consumption of specific foods (fruit, vegetables, home-prepared foods, fat, and proteins) may influence cognitive development, it is possible that this could not be sufficient in those children who do not reach the threshold for minimum dietary diversity.

Three studies that analysed the relationship between diet and social cognition found significant positive outcomes [27, 29, 105]. While two had non-significant results [70, 107], both of which did not use the 24-hours-recall method (which is considered to be the gold standard in this area of research [77, 80] and involved samples of mainly stunted children.

In four studies dietary diversity, and the consumption of specific foods (animal sourced food, diary, fruit, vegetables, home-prepared foods, carbohydrates, eggs, and oils), led to significantly more advanced language development [27, 75, 105, 108]. While five studies (involving mainly malnourished or stunted children) found non-significant outcomes related to language development [29, 70]– [71, 74, 107]. The only study that reported a negative significant outcome found that a high fat intake in 24-month-old children was significantly associated with lower expressive language scores [28]. The authors explained this result by specifying that fat consumption contributed 40–60% of total energy in the first 6 months of life and gradually decreased to 30–35% up to 3 years of age [110]; thus, an excessive fat consumption could lead to negative associations with lower language development. The authors also suggested that the high fat consumption could be due to an unhealthy diet, which could lead to poorer development [111]; however, according to this paper, energy and nutrient intakes met the Singaporean Institute of Medicine guidelines [112].

When considering general development, all studies [25, 27, 29, 69, 105, 107] found a positive significant relationship with diet quality except for one [70]. This study found non-significant outcomes in all areas of development involved in this systematic review, possibly because– due to the lack of a standardized version of the ASQ-3 for Uganda (at least not in 2021 when the study was carried out)– data were compared with the US validated version of the instrument which may not adequately represent a low-income population. The study was also carried out in a region of Uganda with a high rate of under-five stunting that was far above the national trend. The other studies found a significant positive relation between general development and both dietary diversity and the consumption of specific foods (fish, fruits, vegetables, eggs, dairy and animal sourced food).

The investigation of the relationship between the complementary feeding approach and child development is still an emerging area of investigation; thus, we could include only three papers in this review. Out of the three studies included, one found that a complementary feeding approach that allows the child to be more independent during mealtimes is related to both language comprehension and production, one that the proportion of self-feeding (a key characteristic of BLW) was positively associated with language production, while the last one found a non-significant relationship between the complementary feeding approach and language development [52, 54]. However, the latter study involved 6–12-month-old children, that were still too young for a proper language evaluation and used a non-validated and relatively scant measure of language development (i.e., mother-reported first word utterance). The association between Baby-Led-Weaning and language development could be due either to the early experience of manipulating and chewing solid food [113–115] or to participating in family meals, in which infants can be exposed to a wide range of vocabulary and possibly to words that are not used in other contexts [116]– [117]. To our knowledge, no study has yet explored the relationship between the complementary feeding approach and the other developmental areas considered in this review; however, the degree of independence and social stimulation involved in the Baby-Led Weaning approach [45–47] could lead to interesting results also in this respect. All the above studies were conducted in ‘WEIRD’ [36] countries, and it would be important to extend this investigation to children growing in the Majority World [37]– [38], as the Baby-Led Weaning approach may become a sustainable method that could represent a protective factor for nurturing infants especially in LMIC, that face the dilemma of food scarcity rather than of food abundance. Sustainable diets have a low environmental impact, respect biodiversity, are culturally acceptable, economically affordable, nutritionally adequate, safe and healthy [118]– [119]. As such, parents using BLW often make healthier and less environmentally costly food choices than parents using PLW, such as prioritising homemade food over commercially-prepared baby food [52]. Additionally, the practice of encouraging infants to eat the same food consumed by the other family members, rather than specially-prepared food, may reduce meal preparation time and food waste.

It is also important to highlight that some limitations were present in studies that reported significant results. These limitations included using only questionnaires to collect data [25, 27, 29, 54, 69, 105, 107, 108], not accounting for confounding factors in the analysis [74], having a relatively small sample size [55, 73], an imbalanced sex distribution [54, 55] and not being longitudinal [25, 27, 29, 54, 72].

Most studies controlled for several covariates during data analysis (with the exception of [74]), which however were not consistent across studies. The most common covariates were parental education (17 studies), child age (13 studies), child sex (11 studies), socio-economic status (9 studies), family composition (number of siblings, birth order, family size) (5 studies), infant anthropometric information (length, weight, BMI) (5 studies). Other less common covariates were home environment quality, household wall type, milk feeding, mother’s age, work status and marital status, child’s gestational age/prematurity, ethnicity, disease comorbidity, sleeping time, parental smoking, family background, caregiving quality and the availability of children’s books and toys.

## Conclusion

Overall, we can conclude that, although there is evidence of some positive associations between diet quality and child development, this does not appear to be generalizable to countries characterized by a high risk of malnutrition. Thus, it is important to promote programs and interventions to improve children’s diets in areas in which the general conditions may prevent positive relationships between diet quality and developmental outcomes from emerging. It is also important to consider that some of these countries used instruments following US validation norms and that this may have altered the outcomes. The evidence concerning the impact of a complementary feeding approach that allows more independence to the infant is promising but still too scant to lead to generalizable results; thus, far more research (such as experimental longitudinal studies involving children from different socio-economic backgrounds) on the long-term impact of the complementary feeding approach on infant development is strongly needed.

This systematic review presents some limitations that derive from its attempt to recruit as many studies as possible. Indeed, the heterogeneity of outcome measures and participants socio-economic and geographical backgrounds did not allow to reach definitive conclusions, although it provided a wider picture of the importance of diet quality and complementary feeding approach for a healthy psychomotor development in the first years of life.

## Other Information

### Registration and Protocol

This systematic review protocol has been registered in the international prospective register of systematic reviews (PROSPERO) and is publicly accessible under the registration number: CRD42024512876. We adhered to the PROSPERO protocol as much as possible, though some small changes were made. We added considerations about the “general development” because it incorporates the areas of development we included in this review (cognitive, language and social cognition) and it allows to have a general idea of infant development. We also included a study [106] that partially involves 4-month-old children (while our original age range was 6-month- to 6-year-olds) because it also included children who fit our definition. In the process of writing this review, CF and AG were involved as experts in the field of infant nutrition and provided crucial support and information for its completion.

## Data Availability

No datasets were generated or analysed during the current study.

## Key References

- World Health Organization. (2023). WHO Guideline for complementary feeding of infants and young children 6–23 months of age.
  - These guidelines are an important reference point for the study of the weaning phase, as they provide recommendations on best practices regarding milk feeding, age of introduction of complementary foods, dietary diversity, unhealthy foods and beverages consumption, nutrient supplements and responsive feeding.
- Farrow, C., Blissett, J., Islam, S., Batchelor, R., Norman, R., Webber, C.,… Shapiro,L. (2024). Approach to Complementary Feeding and Infant Language Use: An Observational Study. Maternal & Child Nutrition, 21(1), e13762. 10.1111/mcn.13762
  - This paper is to be considered of importance since it provides, for the first time, evidence of a positive relation between infant self-feeding and language development.
- Toh JY, Cai S, Lim SX, Pang WW, Godfrey KM, Shek LP, Tan KH, Yap F, Lee YS, Chong Y, Eriksson JG, Birit BFP, Broekman, Rifkin–Graboi A, Chong MF. Nutrient trajectories during infancy and their associations with childhood neurodevelopment. Eur J Nutr. 2023;62(6):2429–39. 10.1007/s00394-023-03164-2.
  - This paper is of importance since it shows a positive relation between diet and child development in a non-Western country, demonstrating the importance of including papers from all countries in reviews on this topic.

## Footnotes

1 Defined as “*the process by which individuals develop the ability to monitor*,* control*,* and predict the behavior of others*” [66].
